# Supplementary material for: Analysis of Promoter Methylation of the Bovine FOXO1 Gene and Its Effect on Proliferation and Differentiation of Myoblasts
Source: Animals (Basel). 2023 Jan 16;13(2):319. doi: 10.3390/ani13020319 (PMC9854826; doi:10.3390/ani13020319)
Supplement: Supplementary file 1 [file animals-13-00319-s001.zip › Figure S1. Study on DNA methylation level of FOXO1 gene promoter..pdf]

Supplementary Materials:

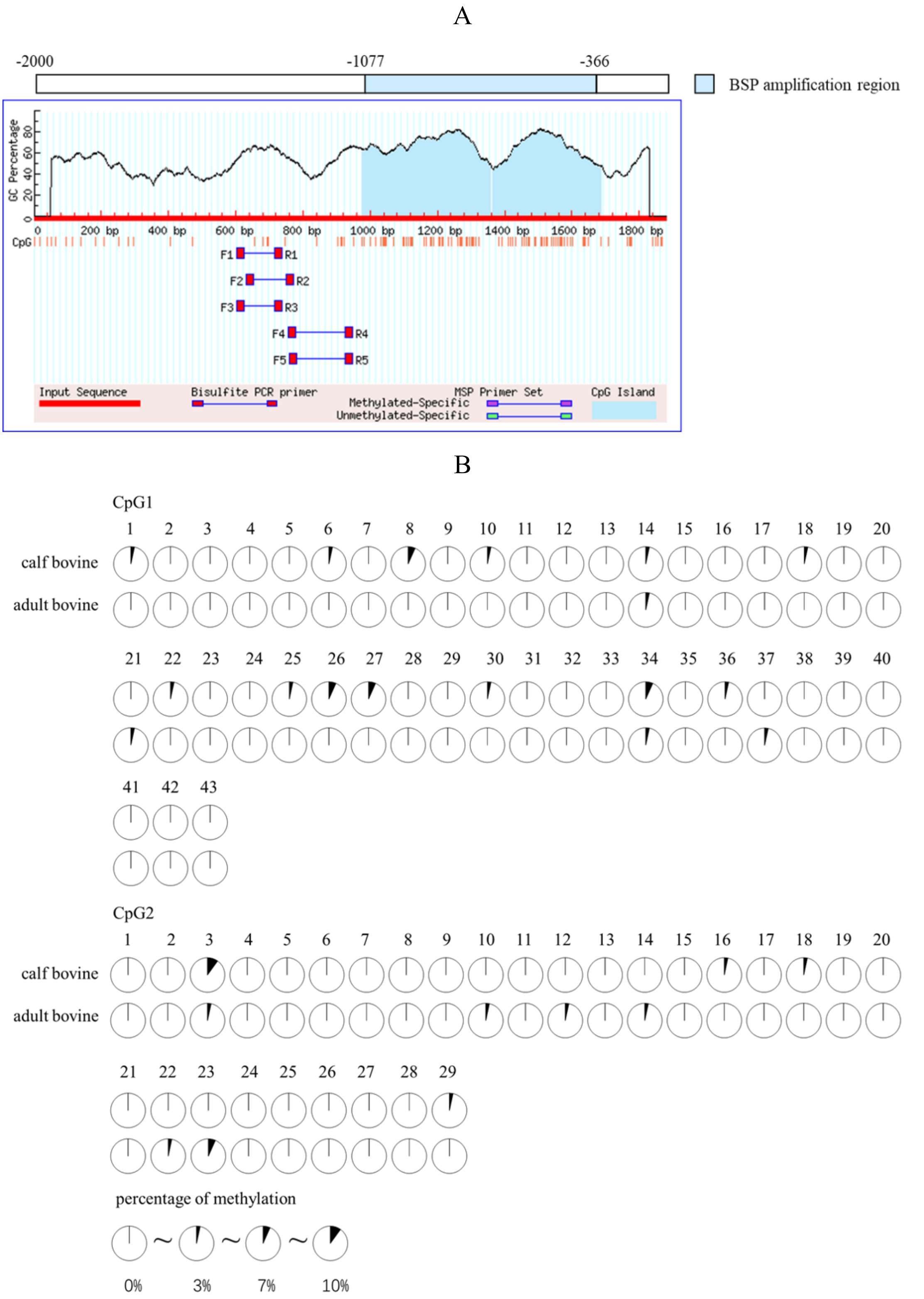

Figure S1. Study on DNA methylation level of *FOXO1* gene promoter. A: The *FOXO1* promoter region was analyzed by online software for the presence of two CpG islands at -366bp to -1077bp. B: The average methylation level of each site in the longissimus dorsi muscle CpG island. Rows indicate different age groups; columns indicate each CpG site with lower overall methylation levels.
