# Supplementary figures and images for: Analysis of Promoter Methylation of the Bovine FOXO1 Gene and Its Effect on Proliferation and Differentiation of Myoblasts
Source: Animals (Basel). 2023 Jan 16;13(2):319. doi: 10.3390/ani13020319 (PMC9854826; doi:10.3390/ani13020319)

## Supplementary Materials:

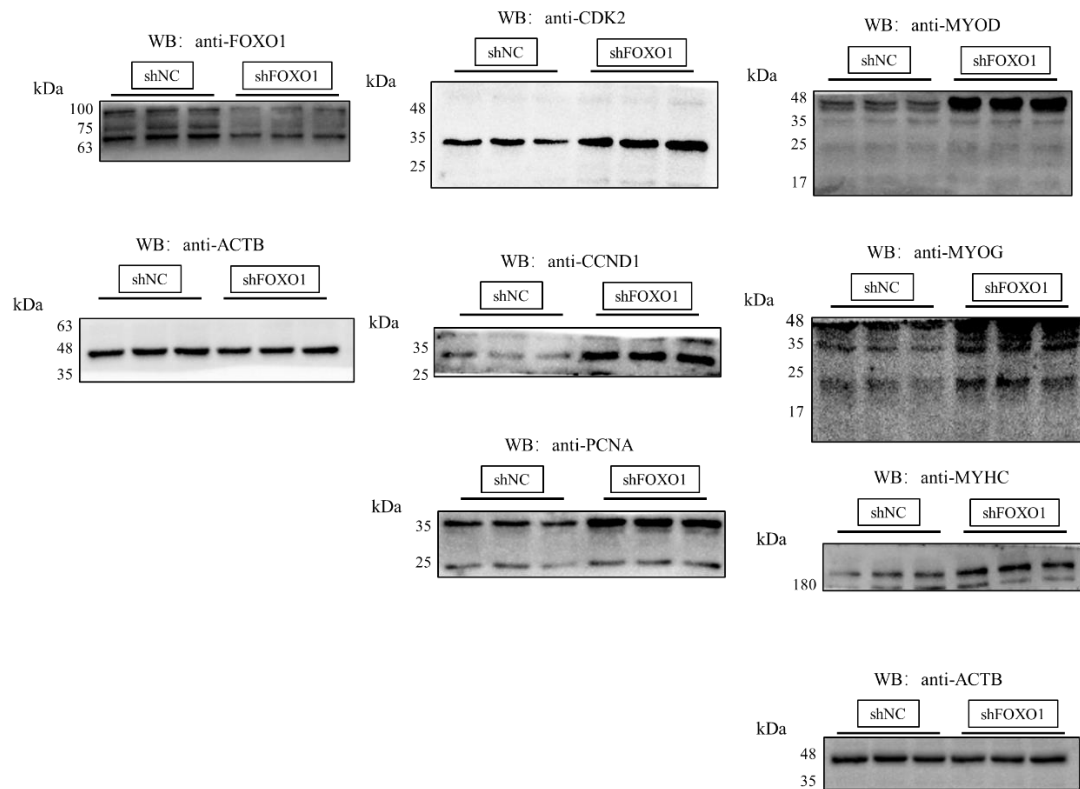

Figure S2. Original western blot figures

Supplement: Supplementary file 1 [file animals-13-00319-s001.zip › Figure S2. Original western blot figures.pdf]
